# Asthma management in the digital age

**DOI:** 10.3389/falgy.2024.1451768

**Published:** 2024-09-03

**Authors:** Ilan Y. Bocian, Andrew R. Chin, Alyssa Rodriguez, William Collins, Sayantani B. Sindher, R. Sharon Chinthrajah

**Affiliations:** Department of Medicine, Sean N. Parker Center for Allergy and Asthma Research, Stanford University School of Medicine, Stanford, CA, United States

**Keywords:** digital tools, asthma, telemedicine, remote patient monitoring, healthcare accessibility

## Abstract

Asthma affects 25 million people in the United States, and its prevalence is increasing. Access to care and adherence to prescribed asthma-treatment programs remain the principal formidable challenges for asthma management. Telemedicine offers substantial opportunities for improved asthma care of patients across the full range of socioeconomic strata. Ever-improving digital tools for asthma assessment and treatment are key components of telemedicine platforms for asthma management. These include a variety of remote patient-monitoring devices, digital inhaler systems, and mobile-health applications that facilitate ongoing assessment and adherence to treatment protocols. Digital tools for monitoring treatment focus on tracking medication use, inhalation technique, and physiological markers such as peak-flow rate and pulse-oximetry. Telemedicine visits allow for elements of assessment via video, approximating or duplicating many aspects of in-person visits, such as evaluating a patient's general appearance, breathing effort, and cough. Challenges remain in ensuring equitable access to these technologies, especially in rural and low-income areas, and in maintaining patient privacy and data security in digital platforms.

## 1 Introduction: the need for telemedicine in asthma care

The COVID-19 pandemic highlighted the need for long-distance communication between physicians and patients, resulting in an explosion in the use of telemedicine. Before the pandemic, telemedicine had been employed only infrequently in healthcare, accounting for roughly 15.4% of healthcare encounters in 2019, but surged to nearly 87% in 2021, corresponding to the height of the COVID pandemic (1).

Telemedicine is defined by the World Health Organization as “the use of information and communication technologies to improve patient outcomes by increasing access to care and medical information” (2). Telemedicine encompasses a wide range of digital tools, including video conferencing, telephone, fax, e-mail, online patient portals, and remote-patient-monitoring devices that are used to gather and transmit patient information for diagnosis and treatment. Asynchronous telemedicine involves communication whereby information is exchanged between the healthcare provider and the patient at different points in time (e.g., through a patient portal), while synchronous telemedicine involves real-time interaction (e.g., via video conference, telephone consultation), where both parties are present simultaneously (2). During the pandemic, these technologies narrowed the gap between patients and healthcare professionals while observing pandemic safety precautions.

Telemedicine has several practical advantages for both patients and physicians, leading to high patient satisfaction (3). The convenience of telemedicine visits promotes a greater willingness on the part of patients to engage the healthcare system for initial and follow-up care, leading to earlier and more-effective preventive-care interventions, including earlier follow-up visits (4). Telemedicine is able to measure clinical parameters while the patients are in their familiar daily environments, providing a more natural perspective on patients’ health conditions. Blood-pressure readings, for example, taken in a patient's home environment tend to provide a more accurate assessment of cardiovascular risk than readings taken in a clinical setting (5). This questions the traditional reliance on in-office diagnosis of hypertension, suggesting that patients’ home environments might be better contexts for certain health evaluations. Advancements in telemedicine and related technologies may, somewhat counterintuitively, foster a closer connection between patient and physician by virtually allowing the physician into the patient's personal space. Moreover, the exposure risk of patients and healthcare professionals to contagion encountered during in-person visits to a healthcare facility is eliminated by telemedicine visits (6–8). The carbon footprint is reduced by obviating the need for travel to and from the healthcare facility (9). Socioeconomic barriers that impede direct and efficient access to healthcare facilities can be mitigated by telemedicine visits (10). Certain populations, such as those in rural settings, are confronted by multiple impediments to healthcare such as geographical and transportation limitations, and a relative paucity of local specialty and subspecialty medical expertise, including allergy and asthma specialists (11). Monetary costs to both the patient and the healthcare facility are reduced (9). Telemedicine visits can be efficiently documented using integrated software systems, such as audio and visual recording, providing unique advantages for patients. These include the capacity for the patient to revisit consultations at subsequent periods for enhanced comprehension and improved adherence to medical recommendations. Overall, recording and playback ability of telemedicine visits has the potential to bolster patient education in disease management and prevention, to relieve patients of the immediate task of memorizing the healthcare provider's one-time recitation of recommendations, and to further empower individuals to actively participate in their healthcare.

While telemedicine holds great promise in bridging the gap in healthcare for individuals living far from medical centers or for whom transportation poses a hardship, it is crucial to consider the potential barriers to telemedicine's adoption by individuals or households in rural and low-income areas. Many people, especially those in rural and low-income areas, have limited or no access to the internet due to reduced or absent service coverage, lack of necessary electronic hardware, or both (12, 13). An even greater contributor to the digital divide are difficulties with digital literacy, which extend beyond low-income areas to include older generations in all economic strata. A comprehensive approach that addresses these challenges is required to ensure that telemedicine's reach is not limited by existing healthcare inequalities. These challenges notwithstanding, the advantages of telemedicine ensure its place in clinical practice, particularly for diseases such as asthma, which are optimally managed through recurring, data-driven patient assessments that are consultative rather than procedural in nature, rendering them well-suited for telemedicine-based care (14–16). This review will focus on telemedical innovations applicable to asthma management.

## 2 Asthma: scope and working definition

The 2021 National Health Interview Survey determined that 20.3 million adults and 4.7 million children have asthma (17). Asthma is the most common chronic disease of children in the United States (18). Asthma, defined as reversible airway obstruction due to chronic endobronchial inflammation, is distinctive among chronic medical conditions in that signs and symptoms may fluctuate in the same patient between acutely severe and absent. Acute symptoms of airway obstruction—wheeze, cough, chest tightness, shortness of breath, and dyspnea on exertion—may be ameliorated by bronchodilating medication. However, since asthma is fundamentally a chronic endobronchial inflammatory disease, sustained control of bronchial inflammation is key to prevention of acute exacerbations that underlie the morbidity and mortality of asthma. Furthermore, inadequate control of bronchial inflammation in asthmatic patients over the long term can lead to irreversible bronchial-wall remodeling, consequent loss of airway elasticity and, thereby, permanent reduction in lung function (19). Therefore, telemedicine approaches would be especially beneficial for asthma in helping to ensure that the disease is assessed and monitored on an ongoing basis in order to optimize respiratory function and minimize asthma exacerbations.

## 3 Adherence to asthma-treatment protocols is improved by telemedicine

Asthma-treatment protocols are multifaceted, encompassing both pharmacologic and non-pharmacologic strategies to alleviate acute symptoms, manage asthma over the long-term, and reduce the risk of asthma exacerbations. Depending on the asthma-severity classification (20), medication protocols typically feature quick-relief, or “rescue,” medications, such as inhaled short-acting β-2-agonists (SABAs), which are usually co-prescribed with anti-inflammatory, or “controller,” medications in the form of inhaled corticosteroid (ICS). While the aforementioned medications form the cornerstone of asthma control, asthma-treatment protocols are often modified to include long-acting β-2-agonists (LABAs), long-acting muscarinic antagonists (LAMAs), leukotriene modifiers, mast-cell stabilizers, theophylline, monoclonal antibody (“biologics”), and subcutaneous allergen-injection immunotherapy (SCIT), to reduce allergic hypersensitivity to asthma-triggering allergens. Such modifications aim to enhance medication adherence and/or to provide additional broncho-relaxant and/or anti-inflammatory efficacy.

Adherence to asthma-treatment regimens has been chronically suboptimal, with an estimated 50% of asthma patients failing to adhere to their prescribed therapeutic regimens (21, 22). Suboptimal or non-adherence to prescribed asthma-inhaler regimens, estimated at 30%–70%, is worse than for many other common chronic diseases including diabetes (35% to 45%) (23), cancer (21%) (24), and hypertension (38%) (24). Adherence to asthma-medication regimens is influenced by several factors, such as beliefs about the chronicity of the illness and the benefits of short- and long-term treatments, socioeconomic status, availability of medical care, and the quality of the patient-health-care-provider relationship (25). Poor adherence can be blamed for 24% of asthma exacerbations and 60% of hospitalizations related to asthma (26, 27). Adherence to inhaled-corticosteroid regimens has been documented to diminish over time, leading to overreliance on short-acting bronchodilators, and increased asthma morbidity, mortality, and healthcare costs (28). This may be due to a pervasive misperception among asthma patients that controller medication is unnecessary in the asthma-asymptomatic state, or that “rescue” bronchodilator medication suffices as monotherapy (29). Furthermore, medication-administration techniques are often faulty, either never having been taught properly from the outset, or needing to be retaught due to decline over time in inhaler technique (24, 30). Patients often unknowingly run out of medication and continue to use an empty inhaler. Many asthma patients, therefore, would benefit from continual tracking of their condition with digital tools in a telemedicine context.

Telemedicine has been documented to improve asthma management. The preponderance of 33 studies analyzed by Almasi et al. examined the effects of medication-reminder alarms or messaging, or of remote asthma-education modules (31). Johnson et al. found a notable increase in inhaled-medication adherence rates with SMS-based reminders (32). Bender et al. reported a 25% higher inhaled-corticosteroid adherence rate among pediatric asthma patients using speech-recognition telephone calls integrated with electronic health records (33). Fedele et al. demonstrated significant improvements in asthma control as measured by the Asthma Control Test (ACT), medication adherence (based on prescription-refill history), and asthma-related quality-of-life (measured by the Pediatric Asthma Quality-of-Life Questionnaire) in adolescents using the AIM2ACT mobile application (34). Collectively, the studies cited by Almasi et al. indicated a cost advantage of telemedicine, albeit with less-robust evidence. Waibel's study of 112 telemedicine visits for allergy and asthma found a reduced need for in-person visits and substantial cost savings, including 200 saved work/school days, $58,000 in travel costs, and 80,000 km not driven (35).

More recently, Portnoy et al. found that telemedicine was as effective as in-person visits in terms of ACT scores and family satisfaction (14), while van den Wijngaart demonstrated more symptom-free days and improved ACT scores with virtual asthma clinics as compared with in-person care (36).

Ong et al. conducted a meta-analysis of 10 studies of the efficacy of telemedicine in pediatric asthma, concluding that telemedicine led to more asthma symptom-free days and a higher rate of well-controlled asthma as compared with in-office care (37). There was no significant difference in emergency-room visits and hospitalization rates between the two groups, owing to a low overall rate of severe asthma in the study populations (37).

A randomized controlled trial is in progress to examine the effect of a telemedicine platform on multiple components of asthma control, including symptoms, personalized action plan, PEF record, inhaler-use adherence, exacerbation frequency, exhaled nitric oxide, anxiety and depression scales, asthma quality-of-life, spirometric parameters, ACT score, and bioanalytic parameters (eosinophil count, IgE) (38).

## 4 Actionable data in telemedical management of asthma

Design, implementation, and optimization of digital tools for asthma management necessitates the careful selection of relevant data such as medical history, vital signs, and respiratory flow, which would ideally be readily accessible electronically to healthcare professionals for each patient with asthma. Utilizing digital tools could also streamline the management process, leading to more-effective treatment strategies and improved patient outcomes. The ensuing sections will describe actionable data in this context.

### 4.1 Medical history

It is essential that telemedicine include conditions that may directly impact asthma management, including quality of past responses to asthma medication(s); atopic disease; non-atopic asthma-exacerbating factors such as tobacco-smoke exposure and other sources of reduced air quality; presence of comorbid conditions such as cardiac disease, gastroesophageal reflux disease, and sleep-disordered breathing; history of medication intolerances and/or medication allergies; language barriers; learning issues; and impediments to obtaining medication. Information regarding the patient's current medications is also critical—namely, medication identification, dosage, frequency, remaining supply, and proper administration technique, particularly with respect to inhaled medications.

### 4.2 Current vital signs

Vital signs are a fundamental component of any medical assessment. Through telemedicine, healthcare providers should ensure that patients or caregivers are trained to accurately measure and report pulse rate, respiratory rate, blood pressure, oximetry, and temperature (3, 5). Real-time, bluetooth-connected electronic stethoscopes are available to record and transmit auscultatory sounds during pulmonary and cardiac examinations (39–41). These data, along with asthma-specific tools, can be used to assess the severity of asthma in real time to inform immediate decision-making regarding the possible need for emergency care and/or adjustments in medication therapy.

### 4.3 Asthma-specific measurements

Objective data, including oxygen saturation, peak-flow rate, forced-expiratory flow rate (FEV1), and cardiac rate and rhythm can be critical in both acute and long-term asthma management. Providers can act on this information by adjusting medication, recommending urgent-care evaluation if needed, or making referrals for additional evaluation. Of note, inspiratory-flow measurement as measured by digital inhaler systems (DIS) shows promise as a marker of physiologic lung function in asthma patients (42).

### 4.4 Protection of patient privacy

In the telemedical management of asthma, safeguarding patient privacy extends beyond general data encryption and secure storage, necessitating a tailored approach to the design of asthma-specific digital tools such as watches and other wearables. These devices, which are integral to tracking asthma-control markers such as respiratory rate or peak flow, are prone to additional risks, such as loss or theft, which could lead to unauthorized access to personal health information (PHI). To mitigate such risks, wearables should be equipped with features such as automatic data encryption, user authentication, and remote data-deletion capabilities, which would allow personal data to be cleared if the device is lost or stolen. Moreover, secure wireless transmission protocols must be in place to protect data as they are transferred from the device to the primary telemedicine infrastructure, which must adhere to HIPAA standards and must be accessible only to professionals with appropriate clearance.

## 5 General telemedical tools for asthma

### 5.1 Telemedicine visits

By leveraging the high-definition audio and video capabilities of today's technology, medical professionals can conduct patient assessments that aim to replicate many of the key features of in-person visits, as described below (43). Patients with asthma can benefit from video visits via a variety of commercially available platforms. Such telemedicine sessions permit a variety of key evaluations to be made with respect to the patient with asthma.

Several fundamental elements of the general physical examination can be appreciated via the video format (44). These include the patient's general appearance; level of alertness; neurocognitive cues (45); skin color (pallor, cyanosis, flushing) (5, 46, 47); vocal quality; and degree of nasal congestion. Effort of breathing can be assessed by observing the patient's chest movements, presence of rapid or shallow breathing, use of accessory muscles of respiration, and presence of nostril flaring (5, 48). Presence, quality, and frequency of cough can be assessed audibly. The presence of audible wheeze can be appreciated. Presence of edema can be visually detected if the patient is able to position the camera to show the area of concern.

Medication reconciliation**—**the process of ensuring that the patient's medication list accurately represents the entirety of the patient's medication regimen, that the same medication that the patient might have obtained from disparate sources will be used at the properly prescribed dosage and frequency, and that medication supplies are in-date and in sufficient quantity**—**can be facilitated during telemedicine visits. Real-time demonstrations of asthma-inhaler technique by the patient can give the physician an opportunity to coach and correct medication-administration technique as needed.

An assessment of the patient's environment would aim to identify potential macro-level sources of allergen or irritant within the patient's surroundings. Visual cues as to the presence of tobacco smoke, stove-heater or fireplace exhaust, pets, or fabric dust-mite-harbors (e.g., rugs, carpeting, tapestries, pillows, bedding) can inform specific recommendations for environmental control measures to reduce exposure levels of atopic or asthmatic triggers (3). While it is acknowledged that certain environmental analyses necessitate sample collection and laboratory evaluation, a virtual walkthrough facilitated by the patient using a smartphone or laptop computer can provide healthcare professionals with insights into the environmental and situational factors of relevance to asthma control. This approach aligns with the practice of some allergy-immunology training programs in which trainees conduct home visits to better understand a patient's real-world context. Technological advancements in optics should enable more-sophisticated remote environmental analysis, further bridging the gap between in-person and virtual environmental assessments.

### 5.2 Remote patient monitoring

Remote patient monitoring (RPM) is a healthcare delivery method that leverages digital technology to observe and analyze patient health data from a distance, typically outside traditional clinical settings (49). RPM is a core feature of telemedicine and includes (1) real-time remote collection of patient-generated data; (2) transmission of those data to a healthcare provider in a different location; (3) analysis of those data by medical staff; and (4) communication of actionable measures to patients (50). The healthcare system has seen substantial progress in disease-specific means of RPM. A wide range of wireless devices that enable RPM in the service of multiple medical specialties has recently become available, including those that measure respiratory rate, heart rate, heart sounds, electroencephalography, electrocardiography, echocardiography, body temperature, body weight, blood pressure, glucose levels, and oxygen saturation or oxygen partial pressures (51–54). Since RPM provides for sustained surveillance of critical clinical measurements, there is great potential for improved levels of cost-effective care of asthma (55). Of great importance is the finding that objective medication-adherence data generated by RPM are substantially more accurate than patients’ self-reportage of their adherence to prescribed asthma-medication programs (56). Healthcare providers would thereby be more accurate in their encouragement of improved medication adherence, which in turn would lead to improved patient outcomes. RPM can be used to apprehend premonitory signs and symptoms of an asthmatic exacerbation in order to enable earlier and more appropriate intervention. As RPM becomes better configured for longitudinal care of patients with asthma, earlier recognition of changes in a patient's respiratory status, and alerting of the healthcare team to such changes, will enable medications and other interventions to be more readily adapted to the evolving episode. For instance, RPM can preemptively detect signs of an asthma attack by monitoring data such as peak-flow measurements and inhaler usage, allowing for prompt and tailored intervention (4). Digital inhaler systems can collect real-time data on medication usage, facilitating immediate adjustments to treatment plans based on precise adherence patterns (4).

On a broader scale, RPM is clearly advantageous in times of limited hospital-based healthcare resources, or when social-distancing or isolation mandates are in place, in temporizing an emergency-room visit when early, remote management would be appropriate. Conversely, RPM can prompt an acute visit to a healthcare facility by detecting critical changes in patient health indicators—such as a significant decrease in peak expiratory flow rate or an increase in rescue inhaler use for asthma patients—thereby allowing for earlier, direct medical intervention before the patient's condition deteriorates further.

## 6 Asthma-specific digital tools

### 6.1 Digital inhaler systems

Digital inhaler systems (DIS) are technologically advanced devices integrated with standard inhalers and inhaler-spacer device systems to monitor and enhance the management of asthma by tracking medication use and inhalation technique (4). These systems provide actionable data to both patients and healthcare providers, for improvement of treatment adherence and, thereby, asthma outcomes. The overarching aim of DIS in the context of asthma management is to leverage technology for the monitoring and recording of inhaler usage and inhalation quality (57). These data can assist healthcare professionals in optimizing asthma treatments and enable patients to self-manage their condition more effectively. Ultimately, these systems strive to improve asthma control, enhance patients’ quality of life, and reduce associated healthcare costs of asthma care (58, 59).

DIS consists of a patient interface and a clinician interface. The utility of these interfaces lies in their ability to record, store, and display key characteristics of inhaler-medication usage. They collectively serve to enhance treatment adherence and effectiveness. The system's ability to identify the medication that is dispensed ensures that the correct medication is administered. DIS offers timestamping the actuation of pressurized-metered-dose and dry-powder inhalers, allowing clinicians to monitor medication-usage patterns and to tailor treatment strategies accordingly. By providing measures of inhalational flow rate and duration of inhalation, DIS records the patient's ability to optimally operate the inhaler. DIS also tracks the duration of inhalation post-actuation and the duration of the post-inhalation breath-hold, key factors that indicate whether the medication is inhaled fully and retained in the lungs sufficiently long for optimal deposition and retention. The inclusion of a measure of medication supply and an alert for medication refill is a significant advantage, as these features help ensure continual treatment by preventing inadvertent interruptions due to lack of medication.

DIS provides readouts accessible to both the patient and to medical staff. This feature fosters a shared understanding of treatment progress, encouraging collaborative patient care. The system is equipped with alerts for scheduled dosing to enhance treatment adherence. DIS incorporates audible coaching elements for proper inhaler technique, such as shaking the canister and orienting the inhaler correctly, thereby boosting treatment efficacy. Environmental-air-quality reports relative to a patient's geographical location that are provided by the system inform patients of potential asthma triggers in their surroundings, allowing them to avoid situations that could exacerbate their asthma symptoms.

Patient-usage data are transmitted to the clinician interface, which enables asthma specialists to view, analyze, and refine key aspects of the prescribed asthma-medication regimen and broader treatment plan that can be used to formulate the asthma specialist's response to the patient (4). The clinician interface of DIS features a comprehensive portal dashboard on which relevant data are displayed for analysis. This functionality allows healthcare professionals to monitor patient progress, adjust treatment strategies as necessary, and make informed, data-based clinical decisions. The myriad capabilities of DIS exemplify the power and potential of digital-health technology in improving asthma management.

Several studies have demonstrated the benefits of DIS**—**namely, improvements in medication adherence, medication-administration technique, and in overall patient outcomes, including a reduced risk of asthma exacerbations. More specifically, usage of inhaled corticosteroid as a controller medication improves with DIS usage, along with a concomitant reduction in the usage of β-2-agonist rescue medication (4, 28). For example, Mosnaim et al. examined the effect on medication adherence of an electronic medication monitor and accompanying smartphone app (Propeller Health) that tracked patients’ use of inhaled corticosteroid and short-acting β-2-agonists (SABA). Baseline asthma-symptom and medication-use data were collected over a 14-day period for 100 patients with uncontrolled asthma, who were then randomized to receive regular clinician feedback on the basis of data gleaned from a DIS, or to a control group who received no feedback. A significant increase of 19% (*P* < .01) in SABA-free days, but a minimal change of −2% (*P* = .40) in ICS use, was observed in the monitored group, while the unmonitored, control group showed an insignificant increase of 6% (*P* = .18) in SABA-free days, but a significant decrease of −17% (*P* < .01) in ICS adherence (28).

### 6.2 Ancillary digital tools for asthma management

The advent of digital (“smart”) tools in healthcare is advancing the quality of remote monitoring of asthma. Smart spacers, for example, not only track medication adherence, but also provide feedback on inhalation technique, which facilitates personalized guidance during telemedicine consultations (60). Hand-held, home-based spirometry as well as peak-flow and FEV1 monitors enable patients to measure lung function on a regular basis, which supplies actionable data for timely adjustment of treatment plans (60–62). A number of studies demonstrate the high accuracy and effectiveness of portable spirometry systems that can be used at home to record and transmit FEV1 and peak-flow rate (61, 63–69). The data transmitted by such devices can be integrated into electronic health records, thereby enhancing continuity of care. Moreover, tools such as smart stethoscopes, along with high-resolution smartphone photography, can be employed for more-precise assessments during telemedicine visits (3). Pulse oximeters (3) and breath-sound monitors (70), such as microphones to detect abnormal breath sounds, can alert healthcare providers to early signs of asthma exacerbations. Hirosawa et al. showed that remote auscultation via an internet-based, real-time, bluetooth-connected electronic stethoscope permits detection and identification of a variety of lung sounds (respiratory rate, normal lung sounds, wheeze, rhonchi, stridor, and coarse and fine crackles) and heart sounds (pulse rate, normal cardiac sounds, S_1_ split, S_2_ split, S_3_-S_4_ gallop, atrial fibrillation, aortic stenosis, aortic regurgitation, mitral stenosis, and mitral regurgitation) at a level of accuracy comparable to that obtained with direct, in-person use of a traditional stethoscope (39). The implementation of smart garments and mattresses is an emerging capability, offering continuous monitoring of respiratory and cardiac parameters, as well as physical activity—all key factors in asthma management (3).

Virtual-reality (VR), a computer-generated visual simulation that offers an interactive learning experience, has potential for patient education, providing an immersive experience that can improve the self-management of asthma. VR simulations can illustrate proper inhaler technique, instruct in the avoidance of known asthma triggers, and guide the acute management of an asthma exacerbation. This triad of educational strategies has the potential to reduce asthma morbidity and mortality, and the consequent financial burden of emergency-department visits and hospital admissions (71).

## 7 Conclusion: assets and limitations of asthma telemedicine in the digital age

The COVID-19 pandemic greatly expanded usage of existing technological healthcare tools, and led to the adaptation of digital technology to the service of asthma care. The advantages of telemedicine-based care for asthma**—**namely, patient access to care; the gathering of actionable, objective data from patients concerning their quality of asthma control, medication usage and adherence; and the ability of patients and healthcare providers to listen and respond to one another in real time**—**may be all the more appreciated in view of the 2 million ER visits and 12.7 million physician visits annually for asthma in the US (72, 73). Digital asthma-monitoring devices can improve adherence to standard asthma-medication regimens, thereby lessening the healthcare costs associated with poor adherence (58, 59). Digital tools coupled with decision-support programs, such as those leveraging artificial intelligence, may also ease the physician's task of sifting through raw data, allowing for more efficient communication of relevant insights to patients.

High-powered longitudinal studies of telemedicine-based care of patients who represent all levels of asthma severity, from mild-intermittent to severe-persistent, are needed in which digital monitoring and data-sharing devices occupy a central role in disease management. These studies should evaluate the effectiveness of such devices in capturing accurate and actionable health metrics, medication-adherence patterns, and asthma-exacerbation prevention, as well as assess satisfaction among patients and healthcare professionals with all aspects of the asthma-telemedicine platform. Research should aim to integrate telemedicine-based tools for asthma into traditional healthcare systems to improve the quality, timeliness, accessibility, equitability, and cost-effectiveness of asthma care (74). Although telemedicine is a valuable tool for allergy practitioners, as it can help to close significant gaps in asthma management, it is not meant to replace in-person medicine. Direct examination—including, but not limited to, otoscopy, ophthalmoscopy, rhinoscopy, rhinolaryngoscopy, chest auscultation, and diagnostic imaging—in the non-virtual setting can provide key objective information that is incompletely obtainable in a pure-telemedicine setting. Moreover, there are elements of the “real-life” physician-patient interaction—not least among them the mutual, direct eye contact that is not possible with current computer-based optical systems—gleaned from the in-person visit that digital mechanisms simply cannot supplant.

These caveats notwithstanding, telemedicine is emerging as a valuable and efficient means of delivering medical care to patients suffering from asthma. Despite the challenges presented by the COVID-19 pandemic, including shifting patient expectations, a shortage of medical practitioners, and financial constraints, telemedicine has proven to be a versatile, reliable, and effective mode of healthcare delivery that has garnered a high degree of patient and healthcare-practitioner satisfaction. Such advantages are integral to the advancement of asthma care in the burgeoning world of telemedicine.
